# The effect of MediYoga on sleep-quality, blood pressure and quality of life among older people with hypertension: study protocol of a pragmatic randomized controlled trial

**DOI:** 10.1186/s12906-025-04846-6

**Published:** 2025-03-20

**Authors:** Signe Stelling Risom, Katrine Vollbrecht Amdi, Maria Wahlström, Trine Bernholdt Rasmussen, Suresh Sharma, Zarina Nahar Kabir, Hanne Konradsen

**Affiliations:** 1 Department of Clinical Medicine, Faculty of Health and Medical Sciences, University of Copenhagen, Copenhagen, Denmark; 2 Department of Cardiology, Herlev and Gentofte University Hospital, Copenhagen, Denmark; 3 Institute of Nursing and Nutrition, University College Copenhagen, Copenhagen, Denmark; 4 Sophiahemmet University, Stockholm, Sweden; 5 College of Nursing, All India Institute of Medical Sciences (AIIMS), Jodhpur, India; 6 Division of Nursing, Department of Neurobiology, Care Sciences and Society, Karolinska Institutet, Solna, Sweden

**Keywords:** Intervention, Cardiology, Nursing research, Yoga

## Abstract

**Background:**

High blood pressure (BP) is a common condition that is estimated to soon affect one third of the worlds’ population. Poor subjective sleep quality is shown to be associated with an elevated risk of high BP, though it is a risk factor that can be modified. Yoga can be used as a complementary therapy to lower BP, but more knowledge on duration and intensity are needed. The overall aim is to test the effect of an online yoga intervention as a complementary therapy. The objectives are to: (1) Test whether online yoga can improve sleep quality, health related quality of life, and lower BP. (2) To explore and describe the implementation of online yoga and the participants’ experiences and perspectives on the intervention in a process evaluation.

**Methods:**

A pragmatic randomized controlled trial will be conducted. The study is described in accordance with the SPIRIT guidelines (Standard Protocol Items: Recommendations for Interventional Trials) and results will be reported following the CONSORT (CONsolidated Standards Of Reporting Trials) guidelines for pragmatic trials. The study is designed as a three arm, randomized superiority trial. Inclusion criteria: ≥65 years old, diagnosed with high BP, speaking and reading Danish, able to consent, no comorbidity that restricts them from participating in the intervention, and daily access to a smartphone or tablet. Participants will be randomly assigned to (i) control group which will be receiving treatment as usual (TAU), or to one of two intervention-groups (ii) performing yoga twice a week for 20 min (20 MIN) or (iii) 40 min (40 MIN) for a total of 10 weeks in addition to TAU. The primary outcome is sleep quality measured by the Pittsburg Sleep Quality Index and secondary outcomes include health related quality of life and BP. Analysis will present differences between groups and be carried out by a statistician blinded to group allocation.

**Discussion:**

The study is grounded in the urgent need to address high BP since pharmacological interventions remain the primary treatment modality, the exploration of non-pharmacological strategies, such as yoga, offers a promising avenue for enhancing patient outcomes in a holistic manner.

**Trial registration:**

ClinicalTrials.gov ID NCT06553820. Protocol version 1. 13th November 2024.

**Supplementary Information:**

The online version contains supplementary material available at 10.1186/s12906-025-04846-6.

## Background

Hypertension is a common condition, and it is estimated that by 2025 one third of the world’s population will have hypertension [1]. Hypertension can have fatal consequences and it is the most important modifiable risk factor for all-cause and cardiovascular morbidity and mortality globally [2]. Diseases associated with longstanding hypertension include myocardial infarction, heart failure, stroke, and dementia [3]. The prevalence of hypertension increases with age, and in Denmark as well as other high-income countries roughly two thirds of the population above the age of 75 years have diagnosed hypertension [4]. With the ageing population rapidly increasing, hypertension will become a major public health issue and initiatives to halt the negative development are in great demand [5].

Pharmacological treatment and control of Blood Pressure (BP) can lower the risk of cardiovascular complications [6]. Several complementary non-pharmacological treatments have been tested, among which dietary modification, physical exercise training, and weight loss have proven most effective, followed by stress reduction, meditation, breathing control, and treatment for depression and anxiety [7]. A literature review concluded that around 40% of older adults are not suited for aerobic exercise due to physical, social, or financial reasons [8] and thus other non-pharmacological options should be explored. Several recent reviews have concluded that breathing exercises can be used as a complementary therapy to lower BP, but also highlighted that more knowledge on duration and intensity are needed to ensure optimal effect [9, 10]. Mindfulness has been shown to improve a range of biopsychosocial conditions, including hypertension, even though more randomized studies are needed [11].

Poor sleep quality is common among older people, as shown in a Swedish cross-sectional study (*n* = 1402) with 70% above 60 years old reporting sleep problems [12]. Poor subjective sleep quality is shown to be significantly associated with an elevated risk of hypertension, though it is a risk factor which can be modified [13]. A recent review concluded that non-pharmacological interventions have the potential to improve sleep quality among older people and increasing evidence supports the long-term positive effects of virtual mindfulness-based interventions on sleep quality [14, 15]. Thus, it is highly relevant to test the effect of easy to perform, non-pharmacological and engageable complementary therapies among older adults with hypertension.

**Fig. 1:**
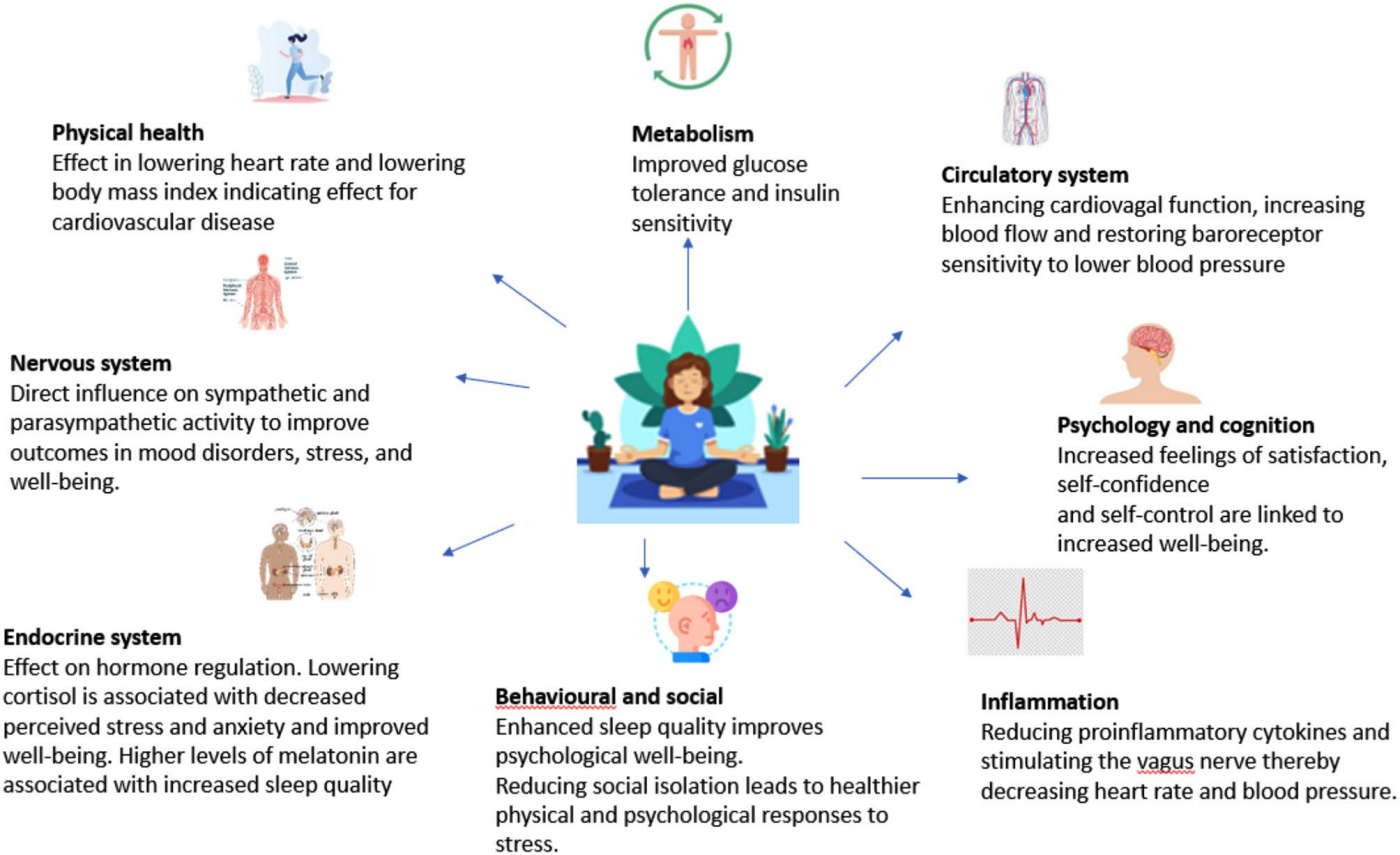
Effects of yoga on health. Figure inspired by McCall [16]

Yoga has been developed and tested and is now considered a non-pharmacological complementary therapy, and its effect can be understood using the model by McCall [16], describing the underlying mechanisms for clinical effects of yoga based on empirical evidence (see Fig. 1). Yoga has proven its suitability as a non-pharmacological complementary treatment among persons with hypertension [17, 18]. There is however limited evidence on the effect of yoga as a complementary therapy among older people and delivered as an online intervention.

## Methods/design

The overall aim of this study is to test the effect of an online yoga intervention as a complementary therapy among persons with hypertension aged 65 years or older. The objectives of the study are to:

1. Test whether online yoga can improve sleep quality, health related quality of life, and lower BP.
2. To explore and describe the implementation of online yoga and the participants’ experiences and perspectives on the intervention in a process evaluation.

The hypotheses are that for people with hypertension ≥ 65 years old, performing online yoga will:

- Improve sleep quality measured by the Pittsburg Sleep Quality Index (PSQI) [19].
- Improve mental health measured by the Hospital Anxiety and Depression Scale (HADS) [20].
- Reduce in BP and improve Health-related Quality of Life (HRQoL) measured by the Short Form-12 questionnaire [21].

### Design

The study is designed as a pragmatic randomized controlled trial [22]. The study is described in accordance with the SPIRIT guidelines (Standard Protocol Items: Recommendations for Interventional Trials) [23, 24] and the results will be reported following the CONSORT (CONsolidated Standards Of Reporting Trials) guidelines for pragmatic trials [25].

Pragmatic trials are valuable when interventions in real-world settings are explored, and the design also facilitates an easier implementation into clinical healthcare if trial results are positive [26].

**Fig. 2:**
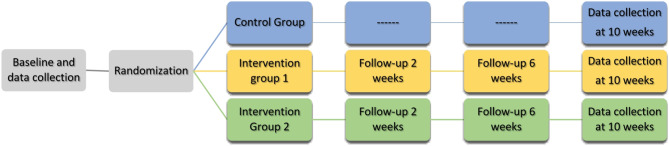
Flow chart of the study

The study is designed as a three arm, randomized superiority trial. Participants will be randomly assigned to one of the three groups (1:1:1) (i) control group which will be receiving treatment as usual (TAU), or to one of two intervention-groups (ii) performing yoga twice a week for 20 min (20 MIN) or (iii) 40 min (40 MIN) for a total of 10 weeks and in addition to TAU (see Fig. 2). Participants receiving the intervention will be invited to participate online in follow-up sessions (after 2 and 6 weeks), where questions regarding the intervention can be addressed and adherence to intervention can be promoted.

### Study setting

The study is anchored in the department of Cardiology at Herlev and Gentofte University Hospital, Denmark.

### Eligibility criteria

Persons are considered eligible for participation if all the following criteria are met: ≥65 years old, diagnosed with hypertension, speaking and reading Danish, able to consent to participate in the study, no comorbidity that restricts them from participating in the intervention, and daily access to a smartphone or tablet. Exclusion criteria are: Starting antihypertensive medication during the intervention, changes in antihypertensive medication (more than 10% increase or reduction in an already used drug or switch to another drug) during the intervention, which includes diuretics, ACE inhibitors, Calcium-blockers, and Angiotensin-2-blockers, participated in mindfulness or yoga regularly at any point during the last two years, having a diagnosis of sleep disorders for example sleep apnea or narcolepsy, and taking insomnia medication regularly. Participants from diverse backgrounds, irrespective of sex and gender, ethnicity, socioeconomic status, or educational background, will be eligible for inclusion in the study.

### Interventions

The intervention is based on yoga and emphasizes active engagement in personal health and therapeutic processes, grounded in the concept of self-management [27].

The clinical evidence of the effect of yoga builds on different practices, traditions, and underlying theories. In this study MediYoga is used, which is originally founded in Sweden in 1997. MediYoga is a standardized, evidence-based yoga intervention intended for use in healthcare, and especially suitable for the management of Non-Communicable Diseases [28]. MediYoga is delivered through a digital platform that offers structured and standardized yoga practices as both therapeutic interventions and self-management tools. The intervention will be conducted in the participants’ home environments via a mobile application. This choice is intended to provide participants with the freedom to choose a comfortable space in which to carry out the intervention. The intervention will be delivered as an add-on to TAU.

Participants in the intervention groups will perform a breathing exercise, light yoga exercises and mindfulness. The positive effects of this combination have been highlighted in a recent review [18]. The verbal and written instructions in the MediYoga programs have been translated into Danish to make it accessible to Danish participants. Active engagement in one’s own health is promoted as participants are encouraged to consistently participate in MediYoga sessions, regularly monitor their blood pressure, and track their sleep quality daily.

Introduction to the intervention will take place in a small group or individually online and led by a MediYoga educated healthcare professional.

### Usual care

All participants will receive TAU irrespective of group allocation. This typically entails pharmacological anti-hypertensive treatment and follow-up according to clinical guidelines and is managed by the participants’ treating physician.

### Outcomes

All data are self-reported by the participants, thus no access to patient records is required.

The primary outcome is sleep quality measured by the PSQI [19]. PSQI is a standardized, self-rated questionnaire that measures different aspects of sleep. It consists of 14 questions concerning factors that might influence sleep quality plus five additional questions that are answered by the bed-partner or roommate. In total, 7 component scores are grouped from the 19 questions, all weighted equally on a 0-to-3 point scale. The components include subjective sleep quality, sleep latency, sleep duration, habitual sleep efficiency, sleep disturbances, use of sleep medications, and daytime dysfunction due to sleepiness. A global score can be calculated from the seven component scores, ranging from 0 to 21. High scores indicate worse sleep quality, and a global score of 5 or higher is characterized as poor sleep quality. Participants are asked to respond thinking about their usual sleep habits for the past 30 days [19]. Sleep quality is measured at Base Line (BL), 10 weeks and at 6 months post intervention.

Secondary outcomes include self-monitored BP using a BP monitor supplied by the research team [29]. The participants receive a guide for measuring BP at home, following the European *Guidelines for the Management of Elevated Blood Pressure and Hypertension* [30]. Participants measure their BP twice weekly with at least two days apart, in the morning, before breakfast and medication. Before measuring, they must have emptied their bladder, avoid smoking for 30 min and rest for 5–10 min. BP is measured three times, with 1–2 min between each measurement.

HRQoL is measured using SF-12 ^21^, a generic tool that includes 12 items covering general health, bodily pain, vitality, social functioning, role functioning, mental health, and physical functioning. Scores can be summarized into a physical component summary score and a mental component summary score. HRQoL is measured at baseline, 10 weeks and 6 months post intervention.

Mental health is measured by measuring symptoms of anxiety and depression using the Hospital Anxiety and Depression Scale (HADS) [20]. The HADS includes 14 items that can be divided into 2 subscales: an anxiety scale and a depression scale, with each scale including 7 items. Each item is rated from 0 to 3 points. Scores from 0 to 7 is considered normal, and scores 8 or higher indicate the presence of a clinical mood disorder [20, 31]. Mental health is measured at baseline, 10 weeks and 6 months post intervention.

Sociodemographic characteristics including age, sex, occupational status, comorbidity, educational level, medication, and family and cohabitation status will be collected via self-report at baseline. At baseline and after 10-weeks, we will inquire about stressful life events (such as a death in the family, divorce, traumatic accidents, etc.). Participants will fill in a dairy, in which they will document adherence to the intervention. In the diary (or submit electronically) they will document (a) BP measured twice a week throughout the intervention period as per instructions, and (b) quality of sleep the previous night rated daily on a VAS scale from 0 to 10. Participants will also be instructed to report changes in antihypertensive medication at the end of the study.

### Participant timeline (see Fig. 3)

**Fig. 3:**
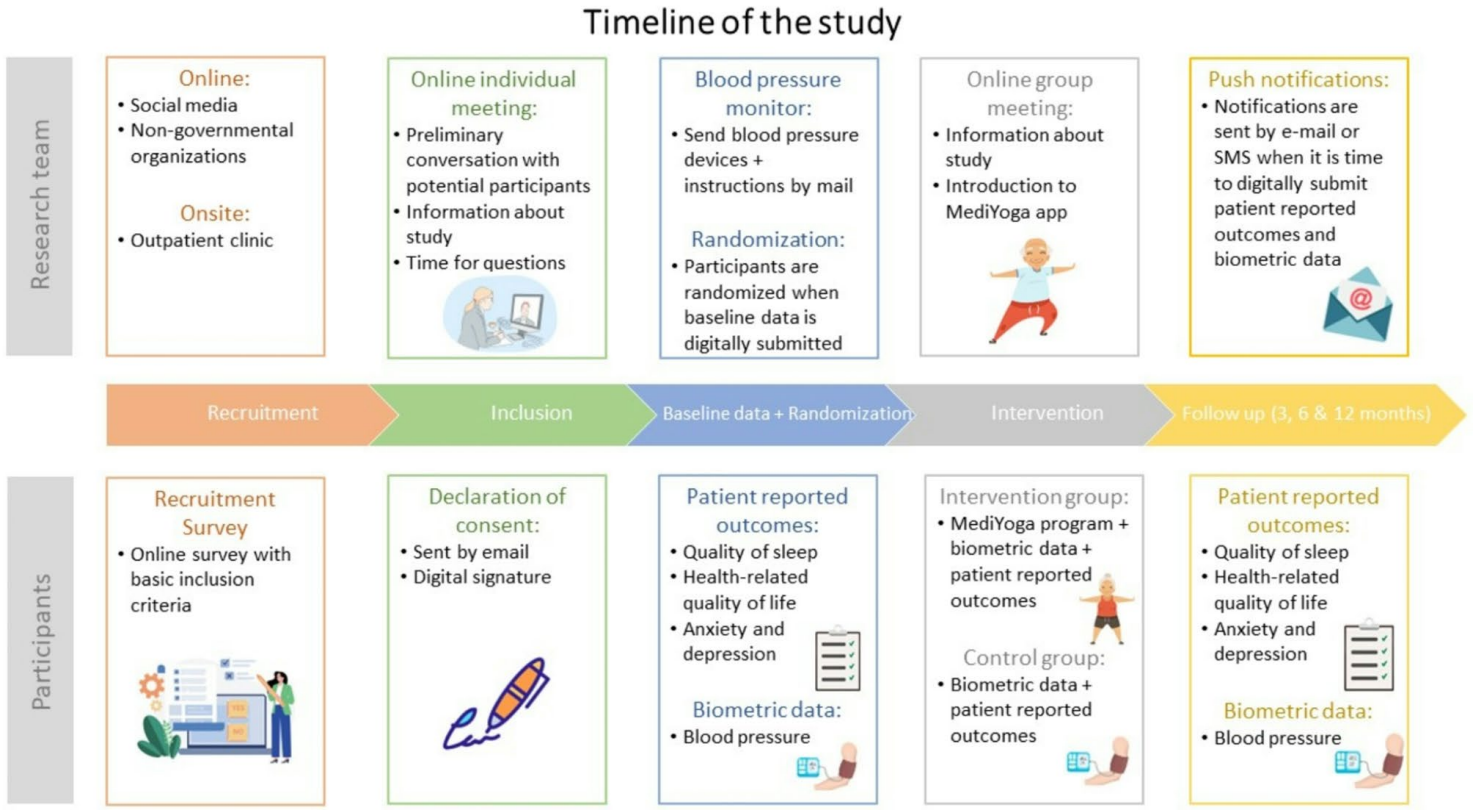
Timeline of the study

### Sample size

Based on prior research including older adults testing mindfulness and meditation in primary care [32] and a clinically relevant difference of 4.4 points on PSQI [33] a sample size has been calculated. Using a power of 80% ad alpha < 0.05, a total of 60 participants (20 in each arm) is needed. Since adherence has been reported to be a challenge in comparable interventions where the patient must be physically active (like exercise training or yoga), we expect an attrition rate of 50% similar to such studies [34, 35]. Considering an attrition rate of 50% it woud mean including 30 in each arm. Considering multiple outcome variables measured in the study (sleep, BP, and quality of life) we consider the design effect (X2) which will mean including 60 participants in each arm, a total sample of 180 participants.

### Recruitment

Recruitment of the participants has begun through social media platforms, Facebook and LinkedIn, as well as through advertisements in cardiology departments and outpatient clinics. A QR code on the advertisement directs potential participants to a webpage where they can complete a form to assess inclusion criteria and provide personal information. Eligible participants automatically receive more detailed participant information about the research and are contacted by phone to address any questions. Following this, a consent form is sent to their secure digital mailbox (which every resident has in Denmark) for electronic signature (See Fig. 3).

### Allocation

Randomization is performed sequentially through REDCap, utilizing six block sizes set at 3, 6, and 9. REDCap is a secure online database designed for building and managing online surveys [36, 37]. The allocation sequence is automatically generated in REDCap, ensuring that the process remains concealed from the investigator until randomization is complete, thereby preventing any potential influence from the researcher on group assignment.

### Blinding

Because of the nature of the intervention, investigators and participants will not be blinded to the intervention group. Statistical analysis will be performed by a statistician blinded to group allocation, and different findings based on varying assignment of the three groups will be formulated before allocation is revealed to ensure high validity of the final results of the study.

### Data collection methods

Surveys and individual QR-code linked to the REDCap platform are used for the collection of data.

### Data management

In this study REDCap facilitates the management of participant information, survey responses, and intervention data.

### Statistical analyses

Statistical analyses of quantitative data will be performed using the software R. Descriptive statistics and inferential statistics will be used. Descriptive statistics include frequencies, percentages, means, and standard deviations. The test of normality will be performed using the Shapiro-Wilk test. Based on the distribution of the data, parametric or non-parametric tests will be applied to test for the significant differences within and between the groups. Drop-out rate will be registered in a flowchart and both per protocol and intent-to-treat analyses will be performed. The level of significance is set as *p* < 0.05. The analysis will be performed by a statistician blinded allocation of the intervention.

### Data monitoring and harms

We expect no harm in relation to participation in the study and no interim analyses are planned. The data manager is a registered nurse, and she will consult a cardiologist located at the hospital where the study is carried out if serious adverse events happen to participants while in the study, to confirm if the serious adverse event could be because of participation in the study if in doubt.

### Process evaluation

The aim of the process evaluation is to explore and describe the feasibility and acceptability of the MediYoga intervention, providing insights into factors like adherence, participant engagement, and implementation challenges. A mixed method approached will be used.

A subset of 15–20 participants from each of the intervention groups will be purposefully selected and invited to participate in individual semi-structured interviews. Additionally, data will be collected from participants’ diaries to analyze their adherence to the intervention and questions about using the app will be addressed using a Likert scale.

By employing both quantitative and qualitative methodologies, the process evaluation seeks to offer a holistic assessment of the intervention’s implementation and effectiveness and allows for a nuanced understanding of how the intervention works in practice and its implications for the participants.

### Analysis

The focus is on identifying and analyzing central themes and patterns within the interviews. Qualitative data will be analyzed using thematic analysis which allows for the exploration and understanding of underlying themes, ideas, and meanings in the interviews, which aligns with the purpose of the process evaluation. The quantitative data will be analyzed with a descriptive analysis where the aim is to provide a clear and comprehensive overview of participants’ adherence and usability of the app.

### Patient and public involvement

Prior to the study’s launch a patient and public involvement group reviewed and provided feedback on participant information, the online advertisement, instructions for downloading the app, instructions for home blood pressure monitoring and the diary. Additionally, they tested the app with the MediYoga sessions. Their feedback has been important in ensuring that the materials are clear, understandable, and relevant to the target group, which has contributed to optimizing the study’s implementation and participant experience.

### Trial status

The first participant was included in the study in November 2024, and the last participant is expected to be included in November 2025.

## Discussion and perspectives

The current protocol outlines a pragmatic randomized controlled trial designed to evaluate the efficacy of an online yoga intervention as a complementary therapy for individuals aged 65 and older with hypertension. The study is grounded in the urgent need to address hypertension, a prevalent condition with significant morbidity and mortality implications, particularly among the aging population. As pharmacological interventions remain the primary treatment modality, the exploration of non-pharmacological strategies, such as yoga, offers a promising avenue for enhancing patient outcomes in a holistic manner.

Hypertension is linked with modifiable risk factors such as physical inactivity. The increasing prevalence of hypertension necessitates innovative strategies that can be feasibly integrated into existing healthcare frameworks. Yoga, with its multifaceted approach encompassing physical postures, breathing exercises, and mindfulness, aligns well with the holistic needs of this demographic.

The primary outcome of this study is the improvement in sleep quality, a critical factor given the high prevalence of sleep disturbances among older adults. Poor sleep quality is intricately linked with hypertension, and improving sleep through yoga could offer dual benefits in managing BP and improving quality of life. Secondary outcomes, including mental health improvements, further underscores the potential of yoga to address biopsychosocial dimensions of health. The incorporation of mindfulness elements within the yoga sessions are particularly relevant, given the growing evidence supporting mindfulness-based interventions for improving mental health and sleep quality.

The pragmatic trial design enhances the generalizability of the findings and facilitates potential implementation in routine healthcare practice, should the intervention prove effective. By leveraging digital platforms for delivery, the study also addresses barriers related to accessibility and engagement, providing a scalable solution that can reach a broad audience.

Despite the promising potential of online yoga interventions, several challenges warrant consideration. Adherence to the intervention may be influenced by factors such as technological literacy, motivation, and individual health status. The anticipated attrition rate of 50% highlights the need for strategies to enhance participant engagement and retention. The process evaluation component of the study is crucial in understanding these dynamics, offering insights into participant experiences and potential barriers to adherence.

## Electronic supplementary material

Below is the link to the electronic supplementary material.

Supplementary Material 1

Supplementary Material 2

Supplementary Material 3

Supplementary Material 4

Supplementary Material 5

## Data Availability

No datasets were generated or analysed during the current study.

## Abbreviations

BP: Blood Pressure
CONSORT: CONsolidated Standards of Reporting Trials
HADS: Hospital Anxiety and Depression Scale
HRQoL: Health Related Quality of Life
PSQI: Pittsburg Sleep Quality Index
TAU: Treatment as Usual
