# Supplementary material for: The effect of MediYoga on sleep-quality, blood pressure and quality of life among older people with hypertension: study protocol of a pragmatic randomized controlled trial
Source: BMC Complement Med Ther. 2025 Mar 20;25:109. doi: 10.1186/s12906-025-04846-6 (PMC11927251; doi:10.1186/s12906-025-04846-6)
Supplement: Supplementary file 1 — Supplementary Material 1 [file 12906_2025_4846_MOESM1_ESM.pdf]

Ethical approval document, translated from Danish.

H-24000021 - Yoga and Elevated Blood Pressure

Final Approval.

The decision is made pursuant to Executive Order no. 1338 of September 1, 2020, on the scientific ethical review of health and health data science research projects.

I confirm receipt of the email dated March 15, 2024, in response to the decision of March 4, 2024, which set conditions for the project's approval. The conditions for approval are deemed fulfilled. The project is therefore finally approved.

The approval is valid until February 28, 2027, and covers the following documents:

- **Study Protocol**, version 3, dated March 15, 2024
- **Participant Information**, version 3, dated March 15, 2024
- **Informed Consent Form**, version 1, dated January 4, 2024
- **Advertisement Text**: Social Media Post, version 1, dated December 14, 2023
- **Diary**, version 1
- **Questionnaires approved for use in the study**:
  - HAD
  - PSQI

The approval applies to the notified study sites and the designated principal investigator in Denmark.

The committee is not the authority responsible for data protection regulations. It is assumed that the project will be conducted in compliance with the GDPR and the Danish Data Protection Act.

Initiating the project contrary to the approval may result in fines or imprisonment, cf. Section 41 of the Committee Act.

Amendments

Significant changes to the protocol material during the project must be submitted as amendments to the committee. The changes may only be implemented after the committee's approval, cf. Section 27, subsection 1, of the Committee Act.

Amendments must be submitted electronically via www.drvk.dk/anmeldelse using the assigned notification number and access code.

Significant changes include those affecting participant safety, the interpretation of scientific documentation underlying the project, as well as project implementation or management. Examples include changes to inclusion and exclusion criteria, study design, the number of participants, study procedures, treatment duration, efficacy parameters, responsible investigators, study sites, and written information provided to participants.

If new information causes the researcher to consider altering procedures or discontinuing the study, the committee must be informed.

Adverse Effects and Incidents

Ongoing Reporting

The committee must be immediately notified of any suspected serious, unexpected adverse effects or serious incidents during the project, cf. Section 30, subsection 1, of the Committee Act. Reports should include comments on potential consequences for the study. Only adverse effects and incidents occurring in Denmark must be reported. Notification must occur no later than 7 days after the sponsor or principal investigator becomes aware of the incident.

A form for reporting is available on the National Center for Ethics website. The form and attachments may be submitted electronically using a digital signature.

Annual Reporting

Annually throughout the study period, the committee must receive a list of all suspected serious (expected and unexpected) adverse effects and serious incidents, along with a safety report for the participants, cf. Section 30, subsection 2, of the Committee Act.

Reports must be in Danish or English. A form for reporting is available on the National Center for Ethics website and can be submitted electronically using a digital signature.

Completion

The principal investigator and any sponsor must notify the committee of the project's completion no later than 90 days after it concludes, cf. Section 31, subsection 1, of the Committee Act.

The project is considered completed when the researcher has finished collecting all data for the study.

If the project is terminated earlier than planned, a justification must be submitted to the committee within 15 days of the decision, cf. Section 31, subsection 2, of the Committee Act.

If the project does not commence, this must also be reported to the committee along with an explanation.

The committee requests a copy of the final research report or publication, cf. Section 28, subsection 2, of the Committee Act. Note that it is mandatory to publish both negative, positive, and inconclusive results, cf. Section 20, subsection 1, no. 8, of the Committee Act.

The obligation to report the completion of the study and the final report rests jointly with the principal investigator and any sponsor.

Supervision

The committee oversees that the project is conducted in compliance with the approval, cf. Sections 28 and 29 of the Committee Act.

Signature on the Consent Form

The committee notes that the principal investigator may delegate the obligation to sign the consent form to the person conducting the oral information session. In such cases, a written delegation must be in place at the study site.

Data Protection - Record-Keeping Requirements

Please note that you may be obligated to register the research project in a record.

If you are a researcher employed in the Capital Region of Denmark, you can contact the Research Legal Unit of the Capital Region for assistance. Further information about the record-keeping requirement is available on their website.

If you are not employed in the Capital Region, you can refer to the Danish Data Protection Agency’s guidelines on record-keeping available on their website.

Confirmation of Approval
The Committees on Health Research Ethics in the Capital Region of Denmark hereby confirm that the above research project is approved and registered for completion by February 28, 2027.

Generally, an approved project is valid throughout the approved period, and regular confirmation of approval does not take place. Unless otherwise stated, the approval includes all documents listed in the approval letter. Extensions may be approved upon application.

Under Danish law, all health research conducted in Denmark must be approved by an Ethical Committee and comply with Danish legislation. Note that "GCP" and "ICH-GCP" rules are only partially implemented in Danish law.

For additional inquiries, please contact the Secretariat for the Committees on Health Research Ethics in the Capital Region at +45 3866 6395 or by email at vek@regionh.dk.

On behalf of Committee Chair Lone Graf Stensballe and Committee Member Bjørn Ebdrup.

Kind regards

Copy sent to: Hanne Konradsen
