# Supplementary material for: The effect of MediYoga on sleep-quality, blood pressure and quality of life among older people with hypertension: study protocol of a pragmatic randomized controlled trial
Source: BMC Complement Med Ther. 2025 Mar 20;25:109. doi: 10.1186/s12906-025-04846-6 (PMC11927251; doi:10.1186/s12906-025-04846-6)
Supplement: Supplementary file 2 — Supplementary Material 2 [file 12906_2025_4846_MOESM2_ESM.pdf]

Region Hovedstaden

Center for Sundhed

De Videnskabsetiske Komitéer
Borgervænget 3, st.
2100 København Ø

Signe Stelling Risom
Herlev og Gentofte Hospital
Afdeling for hjertesygdomme
Kildegårdsvej 28
2900 Hellerup

Afsnit Sekretariatet
Telefon 3866 6395
Direkte 3866 6395
Web www.regionh.dk

Journal-nr.: H-24000021

Dato: 08-04-2024

H-24000021 - Yoga og forhøjet blodtryk

Endelig godkendelse.

Afgørelsen er truffet efter lovbekendtgørelse nr. 1338 af 1. september 2020 - lov om videnskabsetisk behandling af sundhedsvidenskabelige forskningsprojekter og sundhedsdatavidenskabelige forskningsprojekter.

Jeg bekræfter modtagelsen af mail af 15-03-2024 som svar på afgørelse af 04-03-2024, hvori der opstilledes betingelser for godkendelsen af projektet.

Betingelserne for godkendelsen anses for opfyldt. Projektet er dermed endeligt godkendt.

Godkendelsen gælder til den 28. februar 2027 og omfatter følgende dokumenter:

- Forsøgsprotokol, version 3, af 15. marts 2024
- Deltagerinformation, version 3, af 15. marts 2024
- Informeret samtykkeerklæring, version 1, af 4. januar 2024
- Annoncetekst: Opslag på sociale medier, version 1, af 14. december 2023
- Dagbog, version 1
- Spørgeskemaer godkendt til anvendelse i forsøget:
    - HAD
    - PSQI

Godkendelsen gælder for de anmeldte forsøgssteder og den anmeldte forsøgsansvarlige i Danmark.

Komiteen er ikke ressortmyndighed for regelsættet om databeskyttelse. Komiteen forudsætter, at projektet gennemføres i overensstemmelse med databeskyttelsesforordningen og databeskyttelsesloven.

Iværksættelse af projektet i strid med godkendelsen kan straffes med bøde eller fængsel, jf. komitélovens § 41.

Ændringer

Foretages der væsentlige ændringer i protokolmaterialet under gennemførelsen af projektet, skal disse anmeldes til komiteen i form af tillægsprotokoller. Ændringerne må først iværksættes efter godkendelse fra komiteen, jf. komitélovens § 27, stk. 1.

Anmeldelse af tillægsprotokoller skal ske elektronisk på www.drvk.dk/anmeldelse med det allerede tildelte anmeldelsesnummer og adgangskode.

Væsentlige ændringer er bl.a. ændringer, der kan få betydning for forsøgspersonernes sikkerhed, fortolkning af den videnskabelige dokumentation, som projektet bygger på samt gennemførelsen eller ledelsen af projektet. Det kan fx være ændringer i in- og eksklusionskriterier, forsøgsdesign, antal forsøgspersoner, forsøgsprocedurer, behandlingsvarighed, effektparametre, ændringer om de forsøgsansvarlige eller forsøgssteder samt indholdsmæssige ændringer i det skriftlige informationsmateriale til forsøgspersonerne.

Hvor nye oplysninger betyder, at forskeren overvejer at ændre proceduren eller stoppe forsøget, skal komiteen orienteres om det.

Bivirkninger og hændelser

Løbende indberetning

Komiteen skal omgående underrettes, hvis der under projektet optræder formodet alvorlige, uventede bivirkninger eller alvorlige hændelser, jf. komitélovens § 30, stk. 1. Indberetningen skal ledsages af kommentarer om eventuelle konsekvenser for forsøget. Det er kun bivirkninger og hændelser forekommet i Danmark, der skal indberettes. Underretning skal ske senest 7 dage efter, at sponsor eller den forsøgsansvarlige har fået kendskab til tilfældet.

Ved indberetning kan anvendes et skema, der findes på Nationalt Center for Etiks hjemmeside. Skemaet med bilag kan indsendes elektronisk ved anvendelse af digital signatur.

Årlig indberetning

Én gang årligt i hele forsøgsperioden skal komiteen have tilsendt en liste over alle formodet alvorlige (ventede og uventede) bivirkninger og alvorlige hændelser, som er indtruffet i forsøgsperioden sammen med en rapport om forsøgspersonernes sikkerhed, jf. komitélovens § 30, stk. 2.

Materialet skal være på dansk eller engelsk.

Ved indberetning skal anvendes et skema, der findes på Nationalt Center for Etiks hjemmeside. Skemaet med bilag kan indsendes elektronisk ved anvendelse af digital signatur.

Afslutning

Den forsøgsansvarlige og en evt. sponsor skal senest 90 dage efter afslutningen af projektet underrette komiteen herom, jf. komitélovens § 31, stk. 1. Projektet regnes i komiteregi som afsluttet, når forsker har færdiggjort indsamlingen af alle oplysninger til projektet.

Afbrydes projektet tidligere end planlagt, skal en begrundelse herfor sendes til komiteen senest 15 dage efter, at beslutningen er truffet, jf. komitélovens § 31, stk. 2.

Hvis projektet ikke påbegyndes, skal dette samt årsagen hertil meddeles komiteen.

Komiteen beder om kopi af den afsluttende forskningsrapport eller publikation, jf. komitelovens § 28, stk. 2. Vi skal i den forbindelse gøre opmærksom på, at der er pligt til at offentliggøre både negative, positive og inkonklusive forsøgsresultater, jf. komitélovens § 20, stk. 1, nr. 8.

Pligten til at indberette afslutning af forsøg og afsluttende rapport påhviler forsøgsansvarlig og en evt. sponsor i forening.

Tilsyn

Komiteen fører tilsyn med, at projektet udføres i overensstemmelse med godkendelsen, jf. komitélovens §§ 28 og 29.

Underskrift på samtykkeerklæringen

Komiteen gør opmærksom på, at forsøgsansvarlig kan delegere sin pligt til at underskrive samtykkeerklæringen til den person, der holder den mundtlige informationssamtale. Der skal i så fald være en skriftlig delegation hertil på forsøgssitet.

Databeskyttelse - fortegnelseskrav

Du skal være opmærksom på, at du kan være forpligtet til at få forskningsprojektet fortegnet.

Er du forsker ansat i Region Hovedstaden, gør du dette ved at rette henvendelse til Forskningsjura i Region Hovedstaden, som er den regionale enhed, der administrerer forskningsfortegnelsen. Du kan læse mere om fortegnelsen og finde kontaktoplysninger på deres hjemmeside.

Er du ikke ansat i Region Hovedstaden, kan du orientere dig om fortegnelseskravet i Vejledning om fortegnelse på Datatilsynets hjemmeside.

Confirmation of approval

The Committees on Health Research Ethics in the Capital Region of Denmark hereby confirm that above research project is approved and registered to be completed by 28th of February 2027.

Generally, an approved project is valid throughout the accepted period, and a regular confirmation of the approval does not take place. Unless otherwise stated, the approval includes all documents listed in the approval letter. Extension of a study might be approved when applied for later.

According to Danish law all health research taking place in Denmark must be approved by an Ethical Committee and must comply with Danish legislation. Please note, that "GCP" and "ICH-GCP" rules are only partially implemented in Danish law.

For additional inquiries, please contact the Secretariat for the Committees on Health Research Ethics in the Capital Region at +45 3866 6395 or by e-mail vek@regionh.dk

På vegne af komiteformand Lone Graf Stensballe og komitemedlem Bjørn Ebdrup.

Med venlig hilsen/ Kind regards,

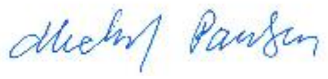

Michael Harbo Paulsen
Cand. Jur

Kopi sendt til: Hanne Konradsen
