# Supplementary material for: The effect of MediYoga on sleep-quality, blood pressure and quality of life among older people with hypertension: study protocol of a pragmatic randomized controlled trial
Source: BMC Complement Med Ther. 2025 Mar 20;25:109. doi: 10.1186/s12906-025-04846-6 (PMC11927251; doi:10.1186/s12906-025-04846-6)
Supplement: Supplementary file 4 — Supplementary Material 4 [file 12906_2025_4846_MOESM4_ESM.pdf]

January 25, 2024

Grant Agreement between the Novo Nordisk Foundation and the Grant Recipient

Dear Hanne Konradsen,

Following peer review, the Novo Nordisk Foundation (the “Foundation”) has the pleasure of awarding you a Grant of up to:

- **DKK 550,000** to support your application with the title **"Yoga and hypertension"** submitted to the call: Project Grants in Nursing Research 2023.
- The grant reference number is **NNF23OC0087238 .**
- The grant period starts on **February 1, 2024** and lasts for 2 year(s) following that date. The grant must be used within this period.
- The administering institution is **Herlev and Gentofte Hospital - Copenhagen University Hospital** .

1. General Terms and Conditions for the Grant

It is important to read this Grant Agreement and the Terms and Conditions for grants from the Foundation carefully.

By signing this Grant Agreement, you confirm that you wish to accept the Grant and that you will comply with the conditions in this Grant Agreement and the Terms and Conditions in force at the time in question.

The Terms and Conditions can be found on the Foundation’s website:

http://www.novonordiskfonden.dk/en/content/conditions-grants

2. Grant payment

A payment plan will be generated in the Foundation’s application and grant management system NORMA before the payment(s) can be requested. Pay-outs may be conditioned upon certain conditions being met.

3. Special conditions applying to the grant

- Reporting on the Grant should be by means of the internet-based reporting tool Researchfish.

- Administrative support constitutes up to 5% of the grant.
- Publication/open access costs may not exceed DKK 25.000 per year.
- Conference participation costs may not exceed DKK 25.000 per year.
- NNF does not support overhead expenses.
- No yearly financial reporting required.
- Final financial reporting required.

4. Comments for the grant

N/A

5. Contact

Questions or comments regarding the grant should be submitted to contact person of the call which you have applied for. An alternative point of contact is the Foundation's secretariat, which can be reached by email at nnfond@novo.dk.

Best regards,
Mads Krogsgaard Thomsen
Chief Executive Officer
Novo Nordisk Fonden

novo nordisk fonden

Signatures

Grant Recipient

I, the undersigned Grant Recipient, am responsible for the veracity and accuracy of the information in the Grant Application and hereby confirm that the funds granted will be applied in accordance with the approved Budget. Furthermore, I confirm compliance with the Novo Nordisk Foundation's standards for good research practice.

DocuSigned by:
Hanne Konradsen
2AB1CACBD553478...

1/25/2024

Signature of Grant Recipient — Date

Name: Hanne Konradsen
Title:

Administrating Institution

I, the authorized signee on behalf of the grant receiving institution, hereby accedes to the Grant Agreement and the Terms and Conditions:

DocuSigned by:
Bodil Ørkild
B967A4D4C901499...

1/25/2024

Signature of signee on behalf of Administrating Institution — Date

Name: Anja Heinsen
Title: chefkonsulent
