# Supplementary material for: The effect of MediYoga on sleep-quality, blood pressure and quality of life among older people with hypertension: study protocol of a pragmatic randomized controlled trial
Source: BMC Complement Med Ther. 2025 Mar 20;25:109. doi: 10.1186/s12906-025-04846-6 (PMC11927251; doi:10.1186/s12906-025-04846-6)
Supplement: Supplementary file 5 — Supplementary Material 5 [file 12906_2025_4846_MOESM5_ESM.pdf]

Kære Hanne Konradsen

Tak for din ansøgning: Yoga mod højt blodtryk og dårlig søvn (ID: 158734).

TrygFonden har behandlet din ansøgning, og vi er glade for at kunne fortælle, at TrygFonden vil støtte projektet med kr. 500.000. Beløbet er inkl. evt. moms.

Anvendelse af bevillingen
Bevillingen er givet under forudsætning af, at projektet gennemføres i sin helhed som fremført i ansøgningen. Såfremt dette ikke er muligt, skal der fremsendes en ny projektbeskrivelse samt budget til TrygFonden til godkendelse.

Udbetaling
Støtte vil blive udbetalt efter dokumentation for udgifter, medmindre særlige forhold gør sig gældende. Når du ønsker dele af eller hele bevillingen udbetalt, skal du ind på vores hjemmeside Tryghed.dk, klik på "Log in", åbne ansøgningen og følge anvisningerne.
Støtte skal udbetales senest en måned efter den slutdato, du har opgjort i ansøgningen. Er dette ikke sket, vil støtten bortfalde. Ændres slutdatoen for projektet skal dette godkendes af TrygFonden pr. mail til info@trygfonden.dk.

Offentliggørelse
Rapporter, pjecer, videnskabelige publikationer, afhandlinger, artikler i tidsskrifter eller anden omtale af projektet skal tydeligt angive, at projektet er gennem-ført med støtte fra TrygFonden. Ønskes der sparring og eller input til formidlingen af projektet, er I velkomne til at tage kontakt til Christian Nørr, TrygFondens dokumentarist, på tlf. 25 45 62 26 eller cn@trygfonden.dk.
Desuden bedes du indsende et eksemplar til fonden, påført ID-nummer.

Evaluering
TrygFonden ønsker at samle og formidle resultaterne fra de projekter, som fonden har doneret midler til. Når projektet er afsluttet, vil du derfor modtage en mail, hvor vi vil bede dig besvare nogle få spørgsmål om projektets afvikling og resultater.

Regnskab

Endeligt godkendt regnskab for projektet skal indsendes til TrygFonden umiddelbart efter projektets afslutning. I tilfælde af at bevillingen kun dækker en del af det ansøgte beløb og projektets samlede budget, skal regnskabet alligevel omfatte samtlige indtægter og udgifter for projektperioden.

Vi gør opmærksom på, at den del af donationen, som evt. måtte dække honorar/lønudgifter, er skattepligtig indkomst.

Tilbagebetaling
Bevillingen skal tilbagebetales, hvis:
• Der ikke aflægges et fyldestgørende underskrevet regnskab.
• Bevillingen eller dele heraf ikke er anvendt i overensstemmelse med betingelserne for bevillingen.

Hvis der er modtaget støtte fra anden side således, at projektet er overfinansieret, vil TrygFonden i så tilfælde kunne anmode om forholdsmæssig tilbagebetaling. I tilfælde af at der efter projektperioden er et uforbrugt beløb, skal dette tilbagebetales.

Eventuelle spørgsmål til donationen bedes rettet til info@trygfonden.dk

Vi ønsker held og lykke med projektet og glæder os til at høre mere om det.

Venlig hilsen
TrygFonden

Rie Odsbjerg Werner
Adm. direktør

Denne mail kan ikke besvares.

TrygFonden smba (TryghedsGruppen smba) · Hummeltoftevej 49 · DK-2830 Virum

Telefon 45 26 08 00 · Fax 45 26 08 01 · info@trygfonden.dk · tryghed.dk
